# Supplementary material for: Causal Relationship between Aging and Anorexia Nervosa: A White-Matter-Microstructure-Mediated Mendelian Randomization Analysis
Source: Biomedicines. 2024 Aug 16;12(8):1874. doi: 10.3390/biomedicines12081874 (PMC11351342; doi:10.3390/biomedicines12081874)
Supplement: Supplementary file 1 [file biomedicines-12-01874-s001.zip › Supplementary Figure/Figure S4/Singe SNP plot of MR analysis for TL to ubm-b-1564.pdf]

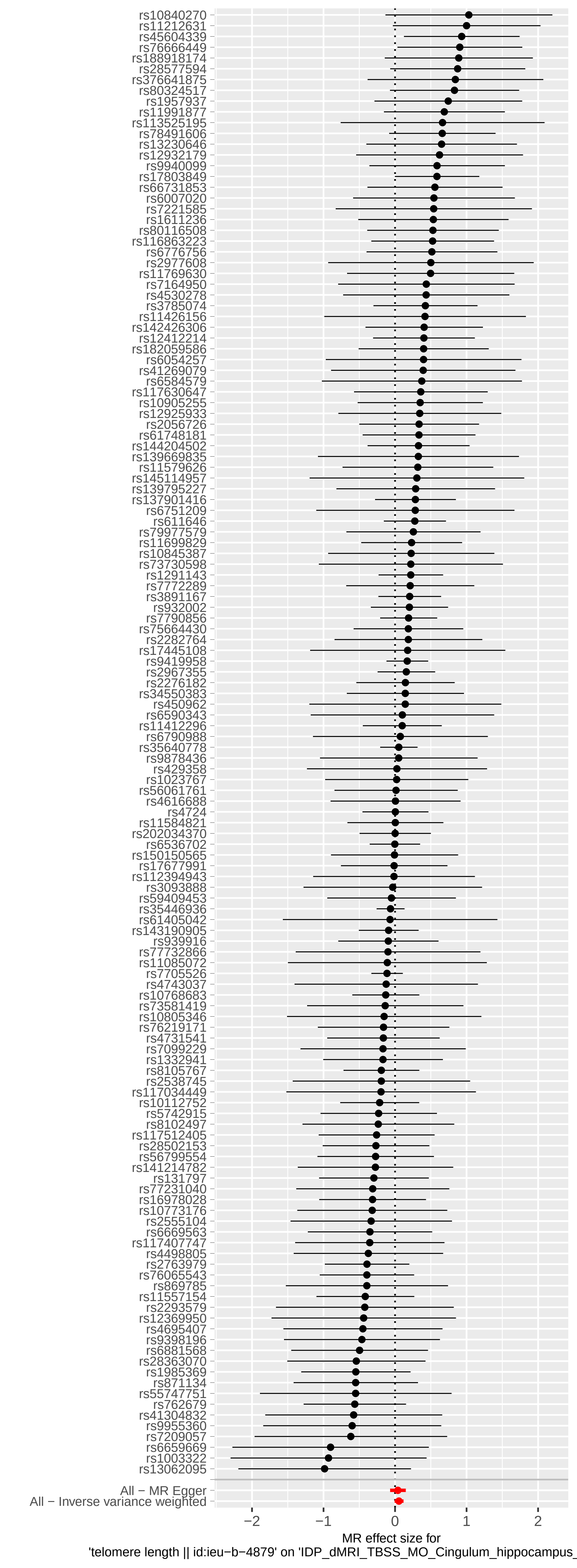
rs10840270
rs11212631
rs45604339
rs76666449
rs188918174
rs28577594
rs376641875
rs80324517
rs1957937
rs11991877
rs113525195
rs78491606
rs13230646
rs12932179
rs9940099
rs17803849
rs66731853
rs6007020
rs7221585
rs1611236
rs80116508
rs116863223
rs6776756
rs2977608
rs11769630
rs7164950
rs4530278
rs3785074
rs11426156
rs142426306
rs12412214
rs182059586
rs6054257
rs41269079
rs6584579
rs117630647
rs10905255
rs12925933
rs2056726
rs61748181
rs144204502
rs139669835
rs11579626
rs145114957
rs139795227
rs137901416
rs6751209
rs611646
rs79977579
rs11699829
rs10845387
rs73730598
rs1291143
rs7772289
rs3891167
rs932002
rs7790856
rs75664430
rs2282764
rs17445108
rs9419958
rs2967355
rs2276182
rs34550383
rs450962
rs6590343
rs11412296
rs6790988
rs35640778
rs9878436
rs429358
rs1023767
rs56061761
rs4616688
rs4724
rs11584821
rs202034370
rs6536702
rs150150565
rs17677991
rs112394943
rs3093888
rs59409453
rs35446936
rs61405042
rs143190905
rs939916
rs77732866
rs11085072
rs7705526
rs4743037
rs10768683
rs73581419
rs10805346
rs76219171
rs4731541
rs7099229
rs1332941
rs8105767
rs2538745
rs117034449
rs10112752
rs5742915
rs8102497
rs117512405
rs28502153
rs56799554
rs141214782
rs131797
rs77231040
rs16978028
rs10773176
rs2555104
rs6669563
rs117407747
rs4498805
rs2763979
rs76065543
rs869785
rs11557154
rs2293579
rs12369950
rs4695407
rs9398196
rs6881568
rs28363070
rs1985369
rs871134
rs55747751
rs762679
rs41304832
rs9955360
rs7209057
rs6659669
rs1003322
rs13062095
All − MR Egger
All − Inverse variance weighted
−2
−1
0
1
2
MR effect size for
'telomere length || id:ieu−b−4879' on 'IDP_dMRI_TBSS_MO_Cingulum_hippocampus_
